# 3-[(*Z*)-Benzyl­idene]-2,3-dihydro-1,5-benzothia­zepin-4(5*H*)-one

**DOI:** 10.1107/S1600536811042991

**Published:** 2011-10-29

**Authors:** V. Sabari, G. Jagadeesan, Raman Selvakumar, Mannickam Bakthadoss, S. Aravindhan

**Affiliations:** a Department of Physics, Presidency College, Chennai 600 005, India; b Department of Organic Chemistry, University of Madras, Chennai 600 025, India

## Abstract

In the title compound, C_16_H_13_NOS, the seven-membered ring adopts a distorted half-chair conformation. In the crystal, mol­ecules are linked by N—H⋯O hydrogen bonds, forming chains running along the *b* axis. The crystal packing is further stabilized by C—H⋯O inter­actions.

## Related literature

For the pharmaceutical properties of thia­zepin derivatives, see: Tomascovic *et al.* (2000 ▶); Rajsner *et al.* (1971 ▶); Metys *et al.* (1965 ▶). For the conformation of thia­zepin derivatives, see: Sridevi *et al.* (2011 ▶).
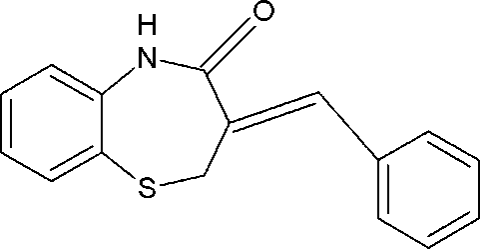


## Experimental

### 

#### Crystal data

C_16_H_13_NOS
*M* *_r_* = 267.33
Orthorhombic, 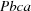

*a* = 10.7711 (9) Å
*b* = 7.8736 (7) Å
*c* = 31.610 (3) Å
*V* = 2680.7 (4) Å^3^
*Z* = 8
Mo *K*α radiation
μ = 0.23 mm^−1^
*T* = 293 K
0.2 × 0.2 × 0.2 mm

#### Data collection

Bruker KappaCCD APEXII diffractometer
Absorption correction: multi-scan (*SADABS*; Bruker, 2004 ▶) *T*_min_ = 0.980, *T*_max_ = 0.990
13085 measured reflections
3306 independent reflections
2643 reflections with *I* > 2σ(*I*)
*R*_int_ = 0.028

#### Refinement

*R*[*F* ^2^ > 2σ(*F* ^2^)] = 0.039
*wR*(*F* ^2^) = 0.112
*S* = 1.04
3306 reflections
180 parameters
H atoms treated by a mixture of independent and constrained refinement
Δρ_max_ = 0.35 e Å^−3^
Δρ_min_ = −0.22 e Å^−3^

### 

Data collection: *APEX2* (Bruker, 2004 ▶); cell refinement: *SAINT* (Bruker, 2004 ▶); data reduction: *SAINT*; program(s) used to solve structure: *SHELXS97* (Sheldrick, 2008 ▶); program(s) used to refine structure: *SHELXL97* (Sheldrick, 2008 ▶); molecular graphics: *ORTEP-3 for Windows* (Farrugia, 1997 ▶); software used to prepare material for publication: *PLATON* (Spek, 2009 ▶).

## Supplementary Material

Crystal structure: contains datablock(s) I, global. DOI: 10.1107/S1600536811042991/bt5678sup1.cif

Structure factors: contains datablock(s) I. DOI: 10.1107/S1600536811042991/bt5678Isup2.hkl

Supplementary material file. DOI: 10.1107/S1600536811042991/bt5678Isup3.cml

Additional supplementary materials:  crystallographic information; 3D view; checkCIF report

## Figures and Tables

**Table 1:** Hydrogen-bond geometry (Å, °)

*D*—H⋯*A*	*D*—H	H⋯*A*	*D*⋯*A*	*D*—H⋯*A*
N—H⋯O^i^	0.872 (18)	1.996 (18)	2.8480 (16)	165.4 (16)
C14—H14⋯O^ii^	0.93	2.57	3.485 (2)	167
C16—H16⋯O	0.93	2.60	3.397 (2)	144

Symmetry codes: (i) 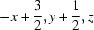; (ii) 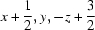.

**Fig. 1.**
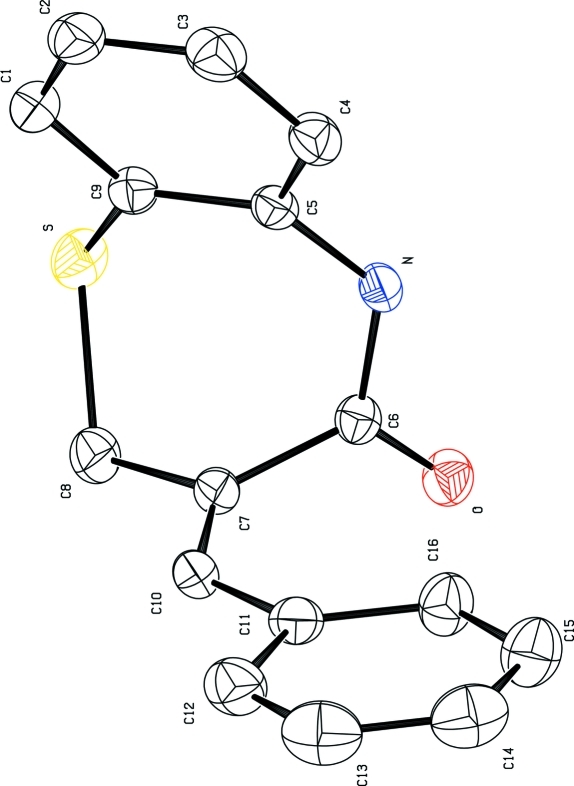
The molecular structure of the title compound, showing 30% probability displacement ellipsoids for non-H atoms. H atoms have been omitted for clarity.

**Fig. 2.**
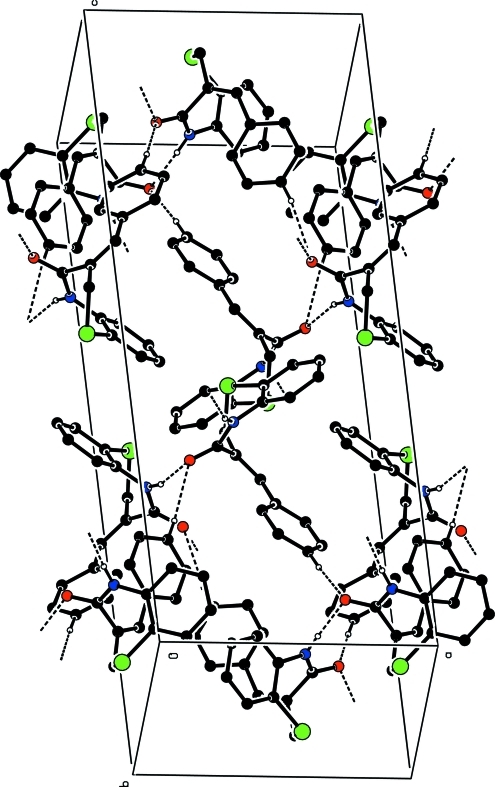
A view of the crystal packing H atoms not involved in hydrogen bonding (dashed lines) have been omitted for clarity.

## supplementary crystallographic information

### Comment

The title compound is used as an intermediate for the synthesis of dosulepin, which is an antidepressant of the tricyclic family. Dosulepin prevents reabsorbing of serotonin and noradrenaline in the brain, helps to prolong the mood lightening effect of any released noradrenaline and serotonin, thus relieving depression. The dibenzo[c,e]thiazepin derivatives exhibit chiroptical properties (Tomascovic *et al.*, 2000). Dibenzo[b,e]thiazepin-5,5-dioxide derivatives possess antihistaminic and antiallergenic activities (Rajsner *et al.*, 1971). Benzene thiazepin derivatives are identified as a new type of effective antihistaminic compounds (Metys *et al.*, 1965). Considering the wide range of biological activities of the thiazepin derivatives, we determined the crystal structure of the title compound. The seven membered thiazepin ring adopts a distorted half-chair conformation (Sridevi *et al.*, 2011). Crystal structure and crystal packing of the molecule were stabilized by intra (C16—H16···O) and Inter (N—H···O, C14—H14···O) molecular interaction.

### Experimental

A mixture of (*z*)-methyl2-(bromomethyl)-3-phenylacrylate (2 mmol) and *O*-aminothiophenol(2 mmol) in the presence of potassium *tert*-butoxide (2,4 mmol) in dry THF (10 ml) was stirred at room temperature for 1 h. After the completion of the reaction as indicated by TLC, the reaction mixture was concentrated and the resulting crude mass was diluted with water (20 ml) and extracted with ethyl acetate (3X20ml). The organic layer was washed with brine (2X20ml) and dried over anhydrous sodium sulfate. The organic layer was concentrated, which provided a crude mass (*Z*)-methyl 2-((2-aminnophyenylthio)methyl)-3-phenylacrylate.The crude product was treated with a catalytic amount of *p*-toluene sulphonic acid (0.4 mmol),in *p*-xylene(10 ml), under reflux conditions for 12 h. After the completion of the reaction as indicated by TLC, the reaction mixture was concentrated under reduced pressure and worked up as mentioned previously, which successfully provide the crude final product. The final product was purified by column chromatography on silica gel to afford the title compound in good yield(71%).

### Refinement

H atoms (except H10 and the amino H atom which were freely refined) were refined with fixed individual displacement parameters [U(H) = 1.2 U_eq_(C)] using a riding model with C-H ranging from 0.93Å to 0.97Å.

### Crystal data

**Table:**

C_16_H_13_NOS	*F*(000) = 1120
*M**_r_* = 267.33	*D*_x_ = 1.325 Mg m^−^^3^
Orthorhombic, *P**b**c**a*	Mo *K*α radiation, λ = 0.71073 Å
Hall symbol: -P 2ac 2ab	Cell parameters from 8725 reflections
*a* = 10.7711 (9) Å	θ = 2.8–29.1°
*b* = 7.8736 (7) Å	µ = 0.23 mm^−^^1^
*c* = 31.610 (3) Å	*T* = 293 K
*V* = 2680.7 (4) Å^3^	Orthorhombic, colourless
*Z* = 8	0.2 × 0.2 × 0.2 mm

### Data collection

**Table:**

Bruker KappaCCD APEXII diffractometer	3306 independent reflections
Radiation source: fine-focus sealed tube	2643 reflections with *I* > 2σ(*I*)
graphite	*R*_int_ = 0.028
Detector resolution: 15.9948 pixels mm^-1^	θ_max_ = 28.3°, θ_min_ = 1.3°
ω scans	*h* = −14→7
Absorption correction: multi-scan (*APEX2*; Bruker, 2004)	*k* = −5→10
*T*_min_ = 0.980, *T*_max_ = 0.990	*l* = −23→42
13085 measured reflections	

### Refinement

**Table:**

Refinement on *F*^2^	Primary atom site location: structure-invariant direct methods
Least-squares matrix: full	Secondary atom site location: difference Fourier map
*R*[*F*^2^ > 2σ(*F*^2^)] = 0.039	Hydrogen site location: inferred from neighbouring sites
*wR*(*F*^2^) = 0.112	H atoms treated by a mixture of independent and constrained refinement
*S* = 1.04	*w* = 1/[σ^2^(*F*_o_^2^) + (0.0544*P*)^2^ + 0.6524*P*] where *P* = (*F*_o_^2^ + 2*F*_c_^2^)/3
3306 reflections	(Δ/σ)_max_ = 0.002
180 parameters	Δρ_max_ = 0.35 e Å^−^^3^
0 restraints	Δρ_min_ = −0.22 e Å^−^^3^

### Special details

**Table:**

Geometry. All e.s.d.'s (except the e.s.d. in the dihedral angle between two l.s. planes) are estimated using the full covariance matrix. The cell e.s.d.'s are taken into account individually in the estimation of e.s.d.'s in distances, angles and torsion angles; correlations between e.s.d.'s in cell parameters are only used when they are defined by crystal symmetry. An approximate (isotropic) treatment of cell e.s.d.'s is used for estimating e.s.d.'s involving l.s. planes.
Refinement. Refinement of *F*^2^ against ALL reflections. The weighted *R*-factor *wR* and goodness of fit *S* are based on *F*^2^, conventional *R*-factors *R* are based on *F*, with *F* set to zero for negative *F*^2^. The threshold expression of *F*^2^ > σ(*F*^2^) is used only for calculating *R*-factors(gt) *etc*. and is not relevant to the choice of reflections for refinement. *R*-factors based on *F*^2^ are statistically about twice as large as those based on *F*, and *R*- factors based on ALL data will be even larger.

### Fractional atomic coordinates and isotropic or equivalent isotropic displacement parameters (Å^2^)

**Table:**

	*x*	*y*	*z*	*U*_iso_*/*U*_eq_	
H10	1.0606 (16)	−0.099 (2)	0.6162 (5)	0.048 (5)*	
H	0.8379 (16)	0.440 (2)	0.6294 (5)	0.042 (4)*	
S	0.90221 (4)	0.20135 (6)	0.529568 (12)	0.04764 (14)	
O	0.73812 (10)	0.16660 (13)	0.64118 (3)	0.0435 (3)	
N	0.88207 (11)	0.35628 (15)	0.61960 (4)	0.0351 (3)	
C8	0.90106 (16)	0.01129 (19)	0.56317 (5)	0.0461 (4)	
H8A	0.8216	−0.0458	0.5605	0.055*	
H8B	0.9653	−0.0665	0.5538	0.055*	
C7	0.92280 (14)	0.05759 (17)	0.60855 (4)	0.0359 (3)	
C6	0.83940 (13)	0.19642 (17)	0.62461 (4)	0.0333 (3)	
C1	1.11685 (15)	0.3856 (2)	0.53349 (5)	0.0445 (4)	
H1	1.1331	0.3404	0.5069	0.053*	
C5	0.98953 (13)	0.39991 (16)	0.59611 (4)	0.0332 (3)	
C2	1.19757 (16)	0.5031 (2)	0.55064 (6)	0.0494 (4)	
H2	1.2669	0.5381	0.5354	0.059*	
C4	1.07215 (15)	0.51731 (19)	0.61325 (5)	0.0432 (4)	
H4	1.0580	0.5614	0.6401	0.052*	
C9	1.01129 (14)	0.33387 (17)	0.55554 (4)	0.0359 (3)	
C16	0.98687 (17)	0.1038 (2)	0.70536 (5)	0.0515 (4)	
H16	0.9123	0.1553	0.6980	0.062*	
C11	1.05193 (15)	0.01041 (19)	0.67530 (5)	0.0412 (3)	
C3	1.17543 (16)	0.5686 (2)	0.59035 (6)	0.0494 (4)	
H3	1.2300	0.6476	0.6019	0.059*	
C13	1.2089 (2)	−0.0450 (3)	0.72861 (7)	0.0690 (6)	
H13	1.2840	−0.0947	0.7362	0.083*	
C14	1.1430 (2)	0.0472 (3)	0.75774 (7)	0.0667 (6)	
H14	1.1730	0.0599	0.7851	0.080*	
C10	1.01213 (15)	−0.01630 (18)	0.63123 (5)	0.0400 (3)	
C12	1.16375 (17)	−0.0644 (2)	0.68770 (6)	0.0555 (4)	
H12	1.2087	−0.1283	0.6683	0.067*	
C15	1.0318 (2)	0.1210 (3)	0.74614 (6)	0.0626 (5)	
H15	0.9867	0.1830	0.7659	0.075*	

### Atomic displacement parameters (Å^2^)

**Table:**

	*U*^11^	*U*^22^	*U*^33^	*U*^12^	*U*^13^	*U*^23^
S	0.0540 (3)	0.0560 (3)	0.0329 (2)	−0.00587 (19)	−0.00722 (17)	−0.00413 (17)
O	0.0376 (6)	0.0446 (6)	0.0483 (6)	−0.0052 (5)	0.0072 (5)	−0.0052 (5)
N	0.0361 (7)	0.0308 (6)	0.0385 (6)	0.0026 (5)	0.0060 (5)	−0.0043 (5)
C8	0.0582 (10)	0.0399 (8)	0.0403 (8)	−0.0077 (7)	0.0035 (7)	−0.0108 (6)
C7	0.0417 (8)	0.0298 (6)	0.0361 (7)	−0.0036 (6)	0.0064 (6)	−0.0030 (5)
C6	0.0345 (7)	0.0355 (7)	0.0299 (6)	−0.0014 (6)	−0.0017 (5)	−0.0031 (5)
C1	0.0500 (9)	0.0433 (8)	0.0401 (8)	0.0036 (7)	0.0095 (7)	0.0038 (7)
C5	0.0341 (7)	0.0283 (6)	0.0372 (7)	0.0031 (5)	0.0010 (6)	0.0020 (5)
C2	0.0441 (9)	0.0434 (8)	0.0607 (10)	−0.0010 (7)	0.0135 (8)	0.0078 (7)
C4	0.0464 (9)	0.0358 (7)	0.0472 (9)	−0.0032 (7)	0.0022 (7)	−0.0056 (6)
C9	0.0388 (8)	0.0343 (7)	0.0345 (7)	0.0022 (6)	−0.0006 (6)	0.0018 (5)
C16	0.0490 (10)	0.0629 (10)	0.0425 (9)	0.0091 (8)	−0.0011 (7)	−0.0004 (8)
C11	0.0413 (8)	0.0367 (7)	0.0457 (8)	0.0010 (6)	0.0020 (7)	0.0063 (6)
C3	0.0447 (9)	0.0383 (8)	0.0652 (11)	−0.0079 (7)	0.0023 (8)	−0.0021 (7)
C13	0.0549 (11)	0.0700 (13)	0.0821 (14)	0.0031 (10)	−0.0195 (10)	0.0156 (11)
C14	0.0707 (13)	0.0733 (13)	0.0561 (11)	−0.0123 (11)	−0.0206 (10)	0.0097 (10)
C10	0.0448 (9)	0.0311 (7)	0.0442 (8)	0.0034 (6)	0.0101 (7)	−0.0017 (6)
C12	0.0491 (10)	0.0524 (10)	0.0649 (11)	0.0090 (8)	0.0010 (8)	0.0075 (8)
C15	0.0678 (13)	0.0746 (12)	0.0454 (9)	0.0002 (10)	−0.0033 (9)	−0.0049 (9)

### Geometric parameters (Å, °)

**Table:**

S—C9	1.7728 (15)	C2—H2	0.9300
S—C8	1.8352 (17)	C4—C3	1.387 (2)
O—C6	1.2326 (17)	C4—H4	0.9300
N—C6	1.3493 (18)	C16—C15	1.384 (2)
N—C5	1.4174 (18)	C16—C11	1.391 (2)
N—H	0.872 (18)	C16—H16	0.9300
C8—C7	1.498 (2)	C11—C12	1.397 (2)
C8—H8A	0.9700	C11—C10	1.473 (2)
C8—H8B	0.9700	C3—H3	0.9300
C7—C10	1.334 (2)	C13—C14	1.371 (3)
C7—C6	1.503 (2)	C13—C12	1.390 (3)
C6—O	1.2326 (17)	C13—H13	0.9300
C1—C2	1.381 (2)	C14—C15	1.381 (3)
C1—C9	1.394 (2)	C14—H14	0.9300
C1—H1	0.9300	C10—H10	0.962 (18)
C5—C4	1.393 (2)	C12—H12	0.9300
C5—C9	1.4037 (19)	C15—H15	0.9300
C2—C3	1.378 (2)		
			
C9—S—C8	102.50 (7)	C5—C4—H4	119.9
C6—N—C5	124.46 (12)	C1—C9—C5	118.98 (14)
C6—N—H	118.7 (11)	C1—C9—S	118.75 (12)
C5—N—H	116.6 (11)	C5—C9—S	122.04 (11)
C7—C8—S	110.78 (10)	C15—C16—C11	120.80 (17)
C7—C8—H8A	109.5	C15—C16—H16	119.6
S—C8—H8A	109.5	C11—C16—H16	119.6
C7—C8—H8B	109.5	C16—C11—C12	117.75 (16)
S—C8—H8B	109.5	C16—C11—C10	125.11 (15)
H8A—C8—H8B	108.1	C12—C11—C10	117.14 (15)
C10—C7—C8	121.42 (14)	C2—C3—C4	120.34 (16)
C10—C7—C6	124.53 (13)	C2—C3—H3	119.8
C8—C7—C6	114.01 (13)	C4—C3—H3	119.8
O—C6—N	121.94 (13)	C14—C13—C12	120.12 (19)
O—C6—N	121.94 (13)	C14—C13—H13	119.9
O—C6—C7	122.26 (13)	C12—C13—H13	119.9
O—C6—C7	122.26 (13)	C13—C14—C15	119.60 (19)
N—C6—C7	115.79 (12)	C13—C14—H14	120.2
C2—C1—C9	120.89 (15)	C15—C14—H14	120.2
C2—C1—H1	119.6	C7—C10—C11	131.02 (14)
C9—C1—H1	119.6	C7—C10—H10	115.1 (11)
C4—C5—C9	119.65 (13)	C11—C10—H10	113.9 (11)
C4—C5—N	118.61 (13)	C13—C12—C11	121.10 (18)
C9—C5—N	121.68 (13)	C13—C12—H12	119.5
C3—C2—C1	119.96 (15)	C11—C12—H12	119.5
C3—C2—H2	120.0	C14—C15—C16	120.63 (19)
C1—C2—H2	120.0	C14—C15—H15	119.7
C3—C4—C5	120.17 (15)	C16—C15—H15	119.7
C3—C4—H4	119.9		
			
C9—S—C8—C7	32.87 (13)	C4—C5—C9—C1	1.0 (2)
S—C8—C7—C10	−126.73 (14)	N—C5—C9—C1	178.07 (13)
S—C8—C7—C6	50.98 (16)	C4—C5—C9—S	−173.34 (11)
O—O—C6—N	0.0 (4)	N—C5—C9—S	3.70 (19)
O—O—C6—C7	0.0 (3)	C8—S—C9—C1	120.08 (13)
C5—N—C6—O	−171.54 (13)	C8—S—C9—C5	−65.54 (13)
C5—N—C6—O	−171.54 (13)	C15—C16—C11—C12	−0.2 (3)
C5—N—C6—C7	8.2 (2)	C15—C16—C11—C10	179.74 (17)
C10—C7—C6—O	−92.01 (19)	C1—C2—C3—C4	−0.1 (3)
C8—C7—C6—O	90.37 (16)	C5—C4—C3—C2	−0.4 (3)
C10—C7—C6—O	−92.01 (19)	C12—C13—C14—C15	−0.2 (3)
C8—C7—C6—O	90.37 (16)	C8—C7—C10—C11	178.60 (15)
C10—C7—C6—N	88.27 (18)	C6—C7—C10—C11	1.1 (3)
C8—C7—C6—N	−89.35 (16)	C16—C11—C10—C7	11.7 (3)
C6—N—C5—C4	−135.93 (15)	C12—C11—C10—C7	−168.36 (17)
C6—N—C5—C9	47.0 (2)	C14—C13—C12—C11	0.7 (3)
C9—C1—C2—C3	1.1 (2)	C16—C11—C12—C13	−0.5 (3)
C9—C5—C4—C3	0.0 (2)	C10—C11—C12—C13	179.57 (17)
N—C5—C4—C3	−177.16 (14)	C13—C14—C15—C16	−0.4 (3)
C2—C1—C9—C5	−1.6 (2)	C11—C16—C15—C14	0.6 (3)
C2—C1—C9—S	172.96 (12)		

### Hydrogen-bond geometry (Å, °)

**Table:**

*D*—H···*A*	*D*—H	H···*A*	*D*···*A*	*D*—H···*A*
N—H···O^i^	0.872 (18)	1.996 (18)	2.8480 (16)	165.4 (16)
C14—H14···O^ii^	0.93	2.57	3.485 (2)	167.
C16—H16···O	0.93	2.60	3.397 (2)	144.

Symmetry codes: (i) −*x*+3/2, *y*+1/2, *z*; (ii) *x*+1/2, *y*, −*z*+3/2.
